# Analysis of the impact of green urban areas in historic fortified cities using Landsat historical series and Normalized Difference Indices

**DOI:** 10.1038/s41598-023-35844-8

**Published:** 2023-06-02

**Authors:** M. Moreno, P. Ortiz, R. Ortiz

**Affiliations:** Department of Physical, Chemical and Natural Systems, University Pablo de Olavide, Utrera Rd. Km 1, 41013 Seville, Spain

**Keywords:** Climate-change adaptation, Sustainability

## Abstract

Urban green areas minimize the negative effects of climatic change and improve the sustainability of historic cities. Despite this, green areas have traditionally been considered a threat to heritage buildings because they cause humidity changes, that accelerate degradation processes. Within this context, this study evaluates the trends in the inclusion of green areas in historic cities and the effects it causes on humidity and conservation of earthen fortifications. To achieve this goal, vegetative and humidity information has been obtained since 1985 from Landsat satellite images. The historical series of images has been statistically analysed in Google Earth Engine to obtain maps that show the means, 25th, and 75th percentiles of the variations registered in the last 35 years. The results allow visualizing spatial patterns and plotting the seasonal and monthly variations. In the decision-making process, the proposed method allows to monitor whether the presence of vegetation is an environmental degradation agent in the nearby earthen fortifications.The analysis of the historic fortified cities of Seville and Niebla (Spain) shows a gradual increase in green areas and an interest in locating them near the earthen fortifications. The impact on the fortifications is specific to each type of vegetation and can be positive or negative. In general, the low humidity registered indicates low danger, and the presence of green areas favours drying after heavy rains. This study suggests that increasing green spaces to historic cities does not necessarily endanger the preservation of earthen fortifications. Instead, managing both heritage sites and urban green areas together can encourage outdoor cultural activities, reduce the impacts of climate change, and enhance the sustainability of historic cities.

## Introduction

From the first mentions of the term cultural sustainability presented by UNESCO at the end of the twentieth century^1^ interest in developing cultural policies that respect both society and the environment has grown exponentially^2–9^. Nowadays, the sustainable management of historic cities is considered a strategic issue and transversal to the objectives of the Agenda for Sustainable Development 2030^10–14^.

Despite this, the phenomena of urban growth increase the level of risk in historic cities and difficult the sustainable management of inhabited cultural environments^15–17^. At the same time, the uncertainty associated with the effects of climate change^18–25^ makes difficult to manage historic cities and preserve its heritage buildings. The increase in adverse climatic events, such as heatwaves and torrential rains^26^ are the main problems that climate change currently poses for the conservation of heritage building^27^. This situation is even more problematic in the case of earthen architecture, which is especially vulnerable to meteorological threats^28,29^.

In this context, urban green areas are important refuges for biodiversity^30,31^ that mitigate the effects of climate change^32–34^. Since there are different types of urban greening^35^, monitoring the dynamics of green spaces is an important prerequisite for improving sustainable urban planning and making it compatible with the conservation of heritage building.

Within this framework, this study aims to identify how the theoretical proposals for the development of sustainable green cities are implemented in historic cities and if the way in which it is done is compatible with the conservation of their cultural heritage. To reach this goal, a methodology based on the use of satellite images is proposed.

Remote sensing makes it possible to study humidity and green surfaces according to their emission spectrum. The satellite images are a set of matrices that store the radiance values ​​recorded by the sensor or the reflectance values ​​obtained by subsequently correcting the image^36,37^. The combined use of satellite images and normalized indices such as Normalized Difference Vegetation Index (NDVI) and Normalized Difference Water Index (NDWI) to identify changes in vegetative surfaces has already been validated by numerous studies^38–44^. Indices were employed in the evaluation of vegetation health, soil humidity and the effects of climate change at international level^45–48^. In recent years, different heritage specialists have applied standardized indices in the prospecting, analysis and management of heritage spaces^49,50^.

Nowadays, statistical analysis of historical series of satellite images^36,51,52^, and processing in the cloud from an Application Programming Interface (API) and a code editor based on JavaScript and/or Python language makes it possible to massively analyse series of satellite images from a personal computer^51,53,54^. This is the reason because, while previous works used standardized indices to analyse single satellite images, recently the analysis of satellite resources on a big data scale has made it possible to use long timescales and to monitor global vegetative changes over time periods of more than 10 years^43,55–57^.

Despite the advantages that this application represents for the sustainable management of heritage urban landscapes, its use is still limited. The elaboration of the indexes from the combination of the bands of the satellite images available in Landsat and Sentinel, it is habitual for the analysis of satellite images one by one^58^ but not for the continuous monitoring of vegetation health. As NDVI values ​​can change after a few days of heavy rain or a drought, the study of a single image does not provide reliable data. However, the availability of continuous data of NDVI values ​​allows one to understand the changes that have occurred in an environment.

While there are different satellite products with estimated weekly continuous NDVI values ​​for analysis at a territorial scale, there is no product for work at an urban scale. The vegetation indices included as a product of the Modis satellite have a pixel size of 1 km, 500 m or 250 m^59^ and do not offer a spatial resolution high enough to allow the study of urban green areas. The products of the NOAA Climate Data Record (CDR) of AVHRR, and NASA Visible Infrared Imaging Radiometer Suite (VIIRS) present a similar resolution adapted to the study of large territories rather than urban environments. As a product derived from Landsat and Modis, the *Global Forest Cover Change* offers a high spatial resolution of 30 m, but with a low temporal resolution of 5 years that does not allow to use this product for real-time management^60^.

To fill this gap, this study proposes and test the statistical use of Landsat images to monitor changes in vegetation and humidity, and obtain maps with seasonal statistical averages and a spatial resolution of 30 m.

The analysis of two historic cities located in the provinces of Huelva and Seville (Spain) allows to identify the changes in green areas during the last 35 years and discuss their influence in the humidity level and conservation of the historic earthen fortifications.

## Materials and methods

### Study area

Two historic cities with earthen fortifications have been selected as study areas. One of them responds to a big city and another one to a small town. However, this method can be applied in any historic city.

**Figure 1:**
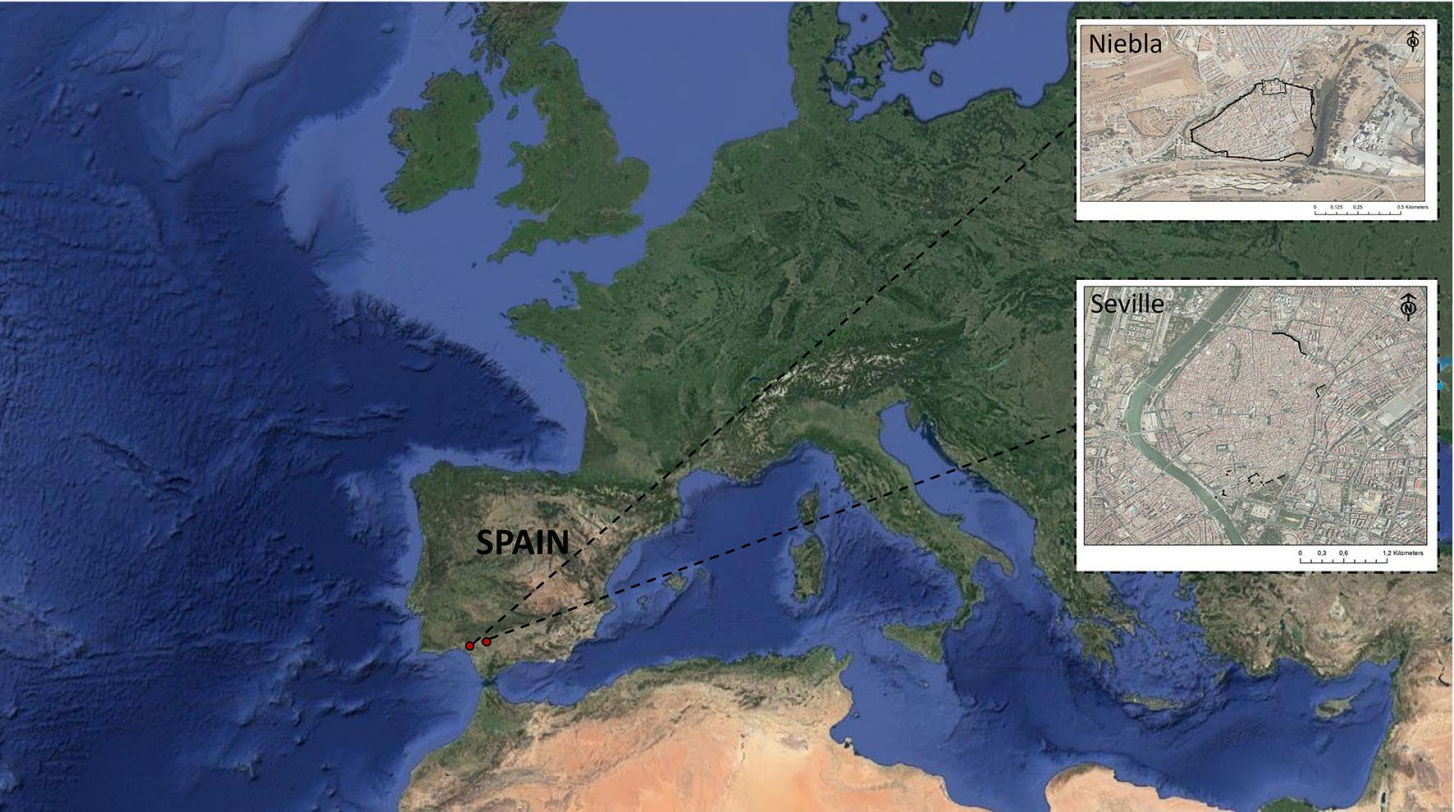
Location and aerial image of the ramparts in Seville and Niebla (Spain, Europe). Base map: Google Maps: Nasa- TerraMetrics, 2021.

The study area is in the south of Spain and includes the cities of Seville and Niebla (Fig. 1). Seville is a provincial capital with approx. 691.000 inhabitants^61^. Located in the valley of the Guadalquivir River, it preserves a historic centre that includes the historic rampart and The Reales Alcázares (or Royal Alcázars). Niebla is a municipality in the province of Huelva with approx. 4.100 inhabitants^61^. It is located next to the ecological corridor of the Tinto River and preserves the entire medieval urban rampart.

Both fortified cities are in the vicinity of two river, the Guadalquivir and the Tinto. The Seville fortifications are surrounded by parks and buildings due to urban growth, while the areas surrounding the Niebla rampart are largely made up of undeveloped areas, shrub and urban forest.

**Figure 2:**
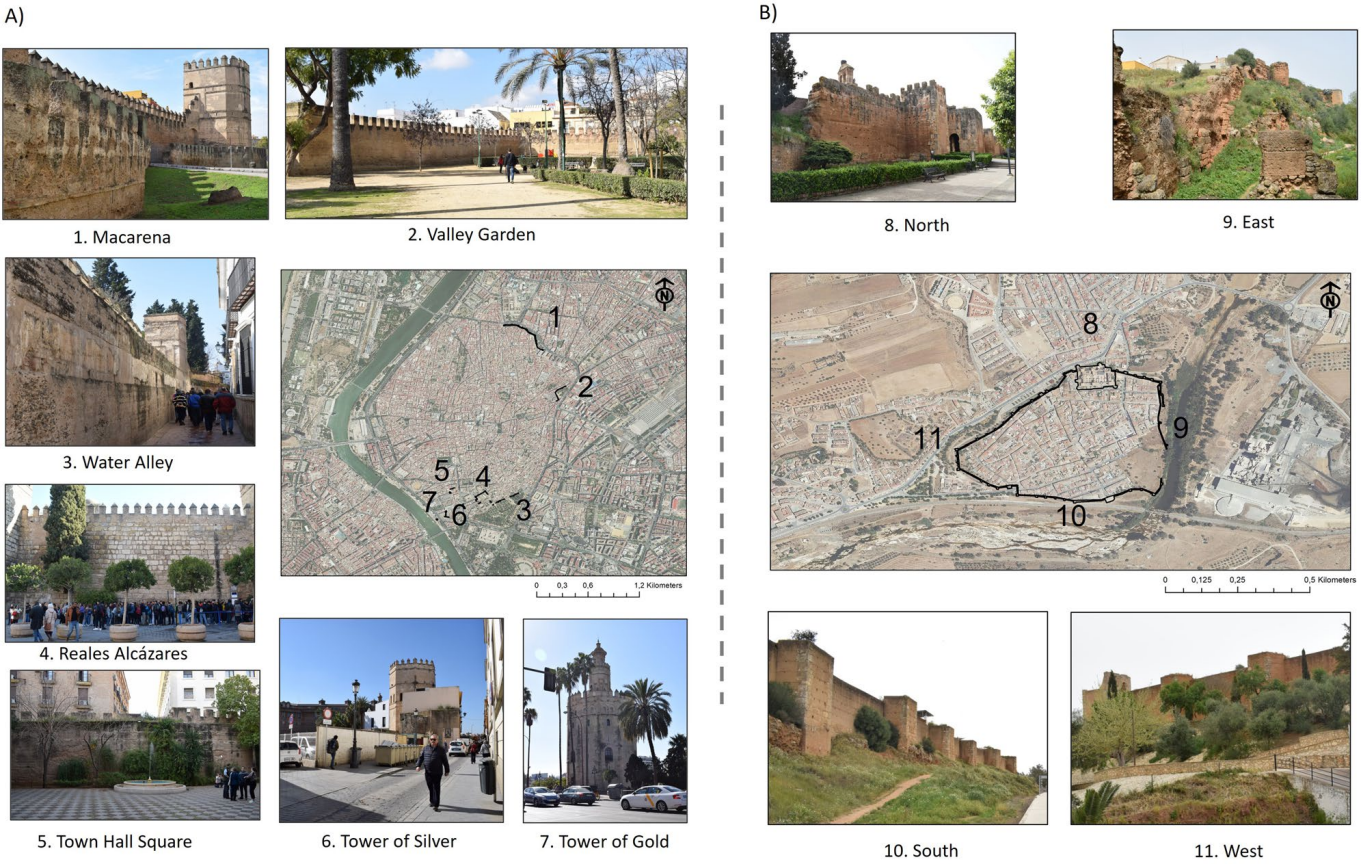
(**A**) Aerial photography and general view of fortifications preserved in public spaces of Seville (Base map: PNOA Orthophotos, 2019). (**B**) Aerial photography and general view of fortifications preserved in public spaces of Niebla. Urban sections are represented in black lines with numbers that correspond to the photographed images (Base Map: PNOA Orthophotos, 2019).

Both fortifications, are significant examples of the Islamic defensive architecture of the southern peninsula. A large part of the preserved remains correspond to Almohad extensions from the twelfth and thirteenth centuries and are mainly rammed earth walls^62–64^ (Fig. 2).

Niebla preserves the entire defensive wall (1.8 km). In the case of Seville, the urban expansion works and the use of more recent buildings as party walls means that approximately 1 of the original 7 km remains visible. Both fortifications have been divided into 10 urban sections in order to be able to relate the preserved remains of the wall with the levels of vegetation, humidity and permeability recorded (Fig. 2) (Seville: 1. Macarena, 2. Jardín del Valle, 3. Callejón del Agua, 4. Reales Alcázares, 5. Cabildo, 6. Torre de la Plata, 7. Torre del Oro; and Niebla: 8. North, 9. East, 10. South and 11. West).

The high vulnerability that earthen walls presents in the presence of water and vegetation^65^ has motivated its choice as a subject for this study. The pathologies that are currently exhibited (moisture areas, detachment and erosion) are the result of environmental factors and the restorations carried out^62,63,65^.

### Google Earth Engine^®^ (GEE^®^)

GEE^®^^66^ has been used for the big data processing of satellite images. GEE^®^ is a cloud computing platform designed for the storage and processing of huge data sets at the petabyte scale. The images used have been obtained from the GEE^®^ catalogue, which is composed of over 5 million images that include the Landsat, Sentinel and MODIS satellite series, vector data sets, digital elevation models and climate data. The satellite images available in the catalogue are ready for use and have already been pre-processed and radiometrically and geometrically corrected.

Once inside the GEE^®^ application, the instructions have been sent via scripts to the GEE^®^ server. GEE^®^ performs server-side processing and returns the results to the user's browser for viewing. The cartography generated can be downloaded, stored in the cloud or shared with other users through the Uniform Resource Locator (url) ^51,53,54^. The scripts designed permit the downloading of the information generated through Google Drive^®^.

### Landsat Images

The Landsat project is a long-term programme that aims to study global changes in the earth's environment. Landsat 1–8 satellites (L1, L2, L3, L4, L5, L7, and L8) provide multispectral imagery from 1972 to the present. It is a series of passive satellites with a polar orbit and a repeating cycle of 16 days^67–69^.

For the study conducted, *Landsat Surface Reflectance* (SR) images with Tier 1 quality from the L5 and L8 satellites have been used, which allow data coverage from 1984 to the present. Bands 3 (0.63–0.69 μm), 4 (0.77–0.90 μm), and 5 (1.55–1.75 μm) have been used From L5, and bands 4(0.63–0.67 μm), 5 (0.85–0.87 μm), and 6 (1.56–1.65 μm) from L8.

The images taken by the Landsat satellite offer complete coverage of the Earth's surface, although images of areas at latitudes of > 65º, extremely arid areas and / or areas that are completely covered by snow may present problems^70^.

### Normalized Indices

Normalized Indices have been used to record the vegetation cover and the humidity level. Normalized Indices are mathematical combinations of two or more spectral bands.

The Normalized Difference Vegetation Index (NDVI) has been used as an indicator of the active photosynthetic biomass. The NDVI is based on the reflectance variations exhibited by chlorophyll, and therefore the biomass, in the red channel, according to its state of health. Healthy vegetation absorbs highly in the blue and red bands and reflects in the green and near infrared (NIR) band. On the contrary, diseased vegetation reflects more in the red band and less in the NIR band. The changes in the spectral curves record modifications in the presence of vegetation from the following equation:

$$NDVI = \frac{NIR - RED}{{RED + NIR}}$$

The normalized values ​​obtained vary between -1 and 1 and can be interpreted as follows: (A) -1 to -0.1: Bodies of Water; (B) -0.1 to 0.2: Rocks, sand, built-up terrain or snow; (C) 0.2 to 0.5 shrubs, grasslands, and cultivated areas; (D) 0.5 to 1: dense vegetation^38,40,41,43^.

The Normalized Difference Water Index (NDWI) has been used as a sensitive indicator to the liquid water levels in the vegetation canopy and complementary to the NDVI. It is based on the variations presented by the NIR and the short-wave mid-infrared (SWIR1) bands, depending on the humidity content of the vegetation cover^38,41,44^. It is used to monitor the presence of bodies of water, droughts and / or the vulnerability of an area of land to fire.

$$NDWI = \frac{{\left( {NIR - SWIR1} \right)}}{{\left( {NIR + SWIR1} \right)}}$$

The normalized values ​​obtained vary between -1 for areas with drier vegetation and 1 for more humid areas. Values ​​below 0 indicate surfaces without vegetation and/or with water masses, values ​​close to 0 indicate water stress and values ​​close to 1 indicate areas with a high presence of humidity in the vegetation^44^.

### Reducers, functions and statistical analysis of Indices

Reducers are summary statistics that take a set of input satellite images and produce a single output. Functions are a compendium of operations that allow applying map algebra to each of the images of a historical satellite series. Functions and reducers are one of the main novelties offered using GEE^®^ and they allow statistically large volumes of satellite images to be worked.

As input data, all the images available for Seville and Niebla with a cloud cover of less than 20% have been used. Using reducers, the seasonality changes, and the five-year percentile 25, percentile 75 and mean of the NDVI and NDWI levels recorded between 1985 and 1990, 1995 and 2000, 2005 and 2010 and 2015 and 2020 have been obtained. Using functions graphs with the annual and interannual variations of vegetation and humidity have been obtained.

The information gathered has been downloaded, classified and interrelated with the vector files of the fortifications analysed using ArcGis^®^, ESRI's desktop GIS software.

## Results

### Patterns and distribution of green areas through the use of reducers

#### Study of the vegetation in the areas surrounding the Niebla fortifications

**Figure 3:**
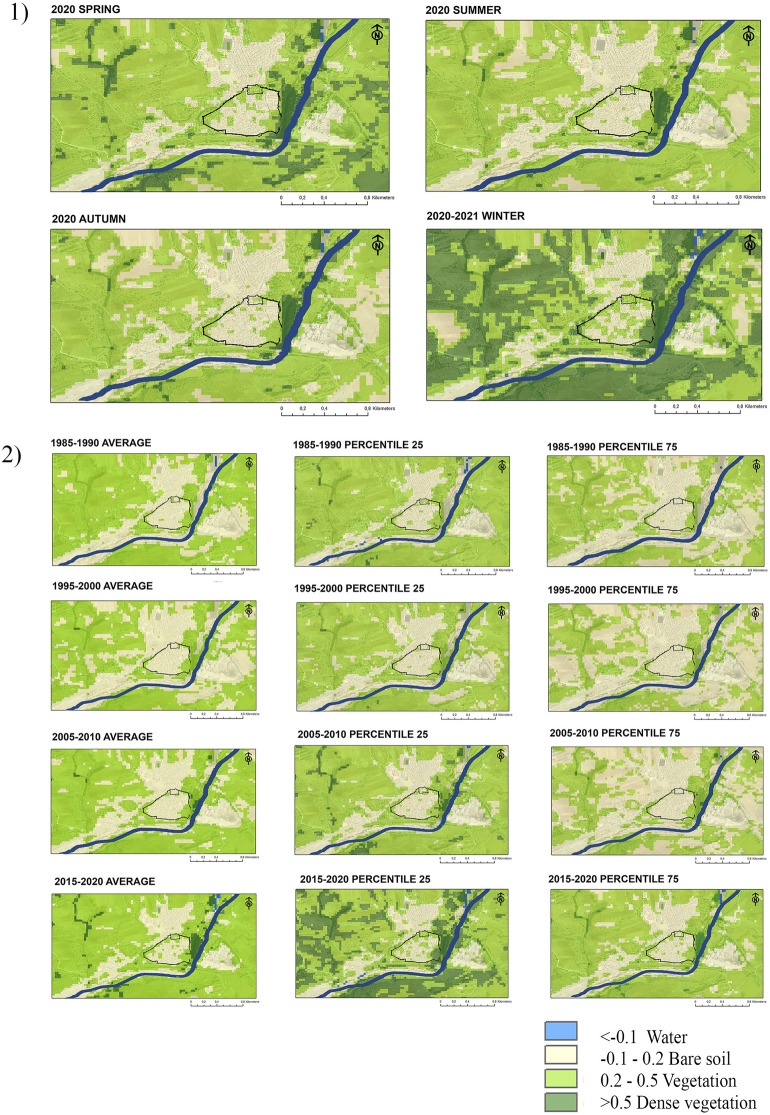
NDVI maps of Niebla: (1) Seasonal averages in 2020 and 2021; (2) Five-year averages and percentiles in 1985–1990, 1995–2000, 2005–2010, 2015–2020. The fortification is represented in black and the riverbed in blue.

The cartography obtained from the statistical analysis of the Landsat image series and the NDVI index ​​allows the identification of changes in the presence of vegetation. Figure 3 shows the strong relationship between vegetative growth and climatic seasonality in the semi-natural environments of Mediterranean climates. Niebla's temperate climate, with a markedly dry and hot period in summer minimises vegetative development in summer. In turn, the greatest development of vegetation in winter (Fig. 3) is caused by the rainfall regime that is distributed mainly in the months of November, December and January^71^. Thus, the NDVI thresholds in Niebla present annual variations of more than 0.6 points in areas covered by deciduous forests, shrub and grasslands.

Vegetation density is also related to the current uses of the soil. Urban soil is delimited in the Fig. 3 by the medieval rampart and appears in yellow except in winter, when the growth of vegetation on the roofs is indicated in green. The non-urbanized areas combine bare soil areas (yellow) and vegetative areas (green and dark green) during all the year.

The Northern, Western and Southern of the rampart (urban Sects. 8,10 and 11) are in public parks, urban forests and rows of pines and poplars. These places have lower NDVI levels (0.2 and 0.5) characteristic of areas where there is control and maintenance. The Eastern side of the municipality of Niebla (urban Sect. 9) is the point that registers the greatest presence of vegetation as of 2015, with levels above 0.5 throughout the year. The dense vegetation identified at this point corresponds to the riverbank and ecological corridor of the Tinto River. Despite being an urban walk area, currently does not have any maintenance. The rampart preserved here it is exposed to environments with high levels of hazard and it is also more vulnerable because or the lack of maintenance and the worse state of conservation (Fig. 2).

In parallel, Fig. 3 shows an increasing trend of vegetative cover in both urbanised and non-urbanised areas since 1985. The comparison of the means makes it possible to identify an increase in dense vegetation in the areas surrounding the municipality from 2005, and also within the municipality from 2015. These areas of dense vegetation are located to the east of the urban core and are adjacent to part of the historic rampart (urban Sect. 9) and to the ecological corridor of the River.

Together with the mean, the percentile maps allow a better understanding of the annual fluctuations. The maps generated from the 25th and 75th percentiles (Fig. 3) correspond to the statistical reduction of the satellite images that registered the 25% lower and the 25% higher levels of NDVI. Their use shows the variations between the months of greater and lesser vegetative growth. The maps generated from the 75th percentiles show better the increase of areas with greater vegetation density in more recent times (2015–2020). The maps generated from the 75th percentiles (Fig. 3), show the reduction of images with a lower level of radiance and a decrease in bare soils during the dry season from 2015. The largest number of bare soil pixels identified between 1995 and 2010 is associated with the occurrence of droughts during the dry months.

#### Study of the vegetation in the areas surrounding the Seville fortifications

**Figure 4:**
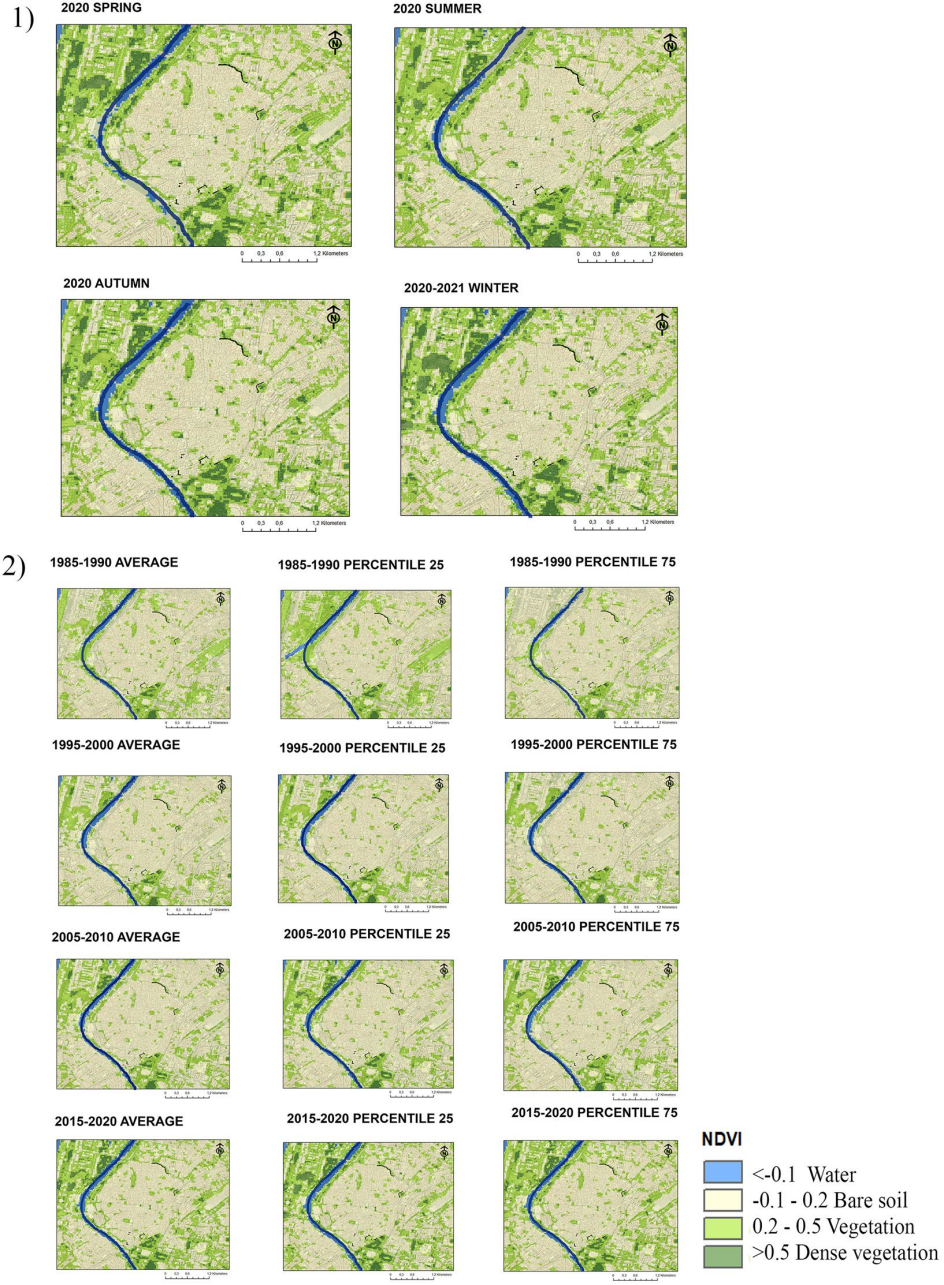
NDVI maps of Seville. (1) Seasonal averages in 2020 and 2021; (2) Five-year averages and percentiles in 1985–1990, 1995–2000, 2005–2010, 2015–2020. The fortifications are represented in black and the riverbed in blue.

Figure 4 show the results obtained for the case of Seville, located 25 km away from Niebla. Due to the expansion of the city, the remains of preserved fortifications have been surrounded by a paved urban environment in which the presence of vegetation corresponds to the development of urban green areas and parks in urban expansions. The high number of pixels that register NDVI levels greater than 0.2 points indicate that Seville is a city with a strong presence of green areas. Seville has numerous historical gardens in which orange trees, palm trees, cypresses, poplars, and jacarandas abound among other species, and it is the city of Andalusia that presenting more surface of green areas per inhabitant^72^.

Regarding the relationship between climate variability and NDVI levels, Fig. 4 enables the observation of the seasonal averages of NDVI recorded in 2020–2021. Seville has a temperate climate with a markedly dry and hot period in summer. Despite the fact that the climates of Seville and Niebla are very similar, in the case of Seville, there is no inter-annual variation in urban NDVI levels. The main causes are the presence of mostly irrigated green areas and the predominance of perennial species.

Figure 4 shows the diachronic evolution in the presence of urban green areas from 1985 to the present. Starting in 2005, there has been a gradual increase in green areas around the historic centre, particularly on the Northwestern bank of the Guadalquivir River and the South Western side of the city. The green areas located inside the historic centre have remained largely unchanged since 1985, with the southern area being that with the highest vegetation density. Despite this, there is a tendency to locate green areas in the surroundings of historic rampart, that appear marked in black in the figure. Although most of the urban sections of fortifications are located in green areas, only one of them is located next to a point of dense vegetation (urban section 3 according to figure 2).

### Patterns and distribution of humidity in green areas through the use of reducers

#### Study of the humidity in the areas surrounding the Niebla fortifications

**Figure 5:**
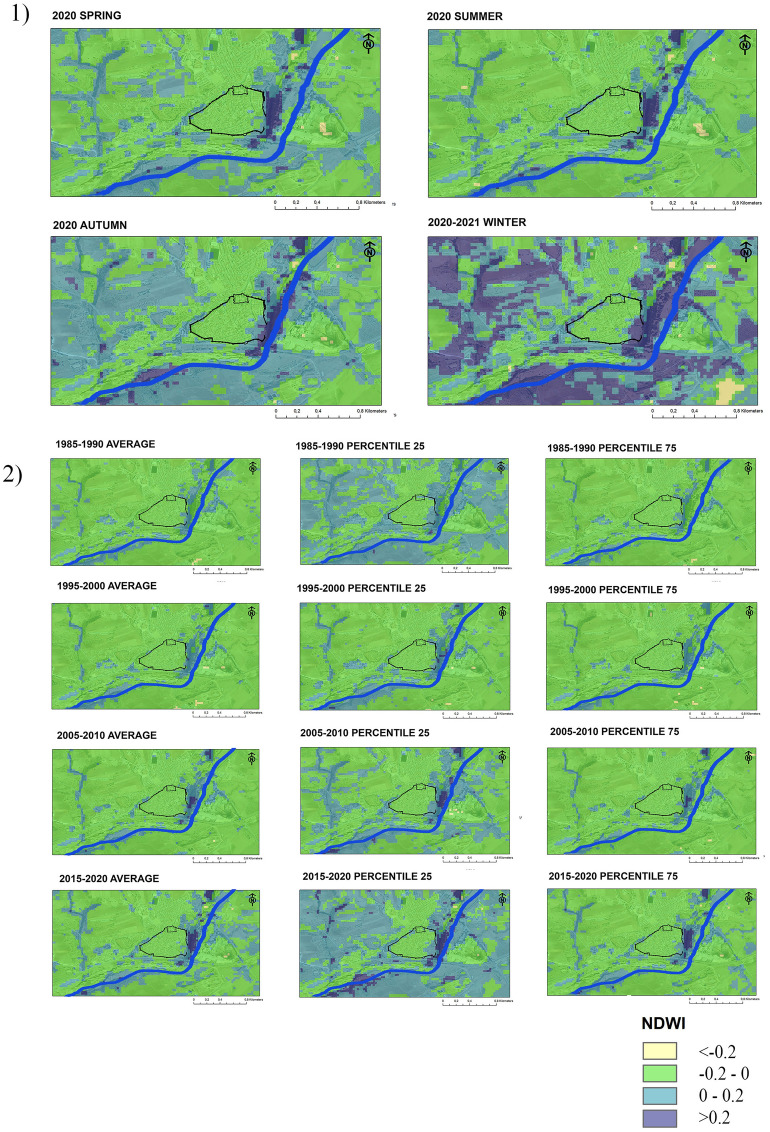
NDWI maps of Niebla. (1) Seasonal averages in 2020 and 2021; (2) Five-year averages and percentiles in 1985–1990, 1995–2000, 2005–2010, 2015–2020. The fortification is represented in black lines and the river channel in blue.

The analysis of the NDWI levels identifies the humidity changes registered by the urban vegetation. As in the case of the NDVI, Fig. 5 show a strong relationship between seasonality and the presence of water in the vegetation. In this case, autumn and winter are the times of greatest humidity, while in summer and spring the high temperatures of the Mediterranean climate cause a sudden drop in humidity.

The areas with a higher presence of humidity are marked in dark blue, and located in non-urbanised spaces, outside the municipality. The eastern side of Niebla, a point with a higher vegetation density and without maintenance, also maintains high levels of vegetative humidity, even in the hottest seasons, especially since 2005 (Fig. 5). The green and light blue areas located within the municipality do not correspond to points of high humidity, and in no case do they present NDWI values greater than 0.2. The low NDWI values ​​indicate that although the vegetation near the rampart is healthy, it does not have a high humidity content in the canopy and the soil surface.

Figure 5 shows the changes recorded in Niebla over the last 30 years, according to the statistical analysis of NDWI. The general trend has been an increase in the presence of humidity around the municipality since 2005, especially on the banks of the Tinto River. The maps generated from the 25th percentile (Fig. 5) show the statistical reduction of the images that registered the greatest presence of water and identify the Southeastern side of the municipality as the wettest area. At the same time, the maps generated from the 75th percentile reflect the decrease in water resources during the driest times. In general, an increase in the humidity of the vegetation is observed, but without reaching dangerous levels (> 0.2).

#### Study of the humidity in the areas surrounding the Seville fortifications

**Figure 6:**
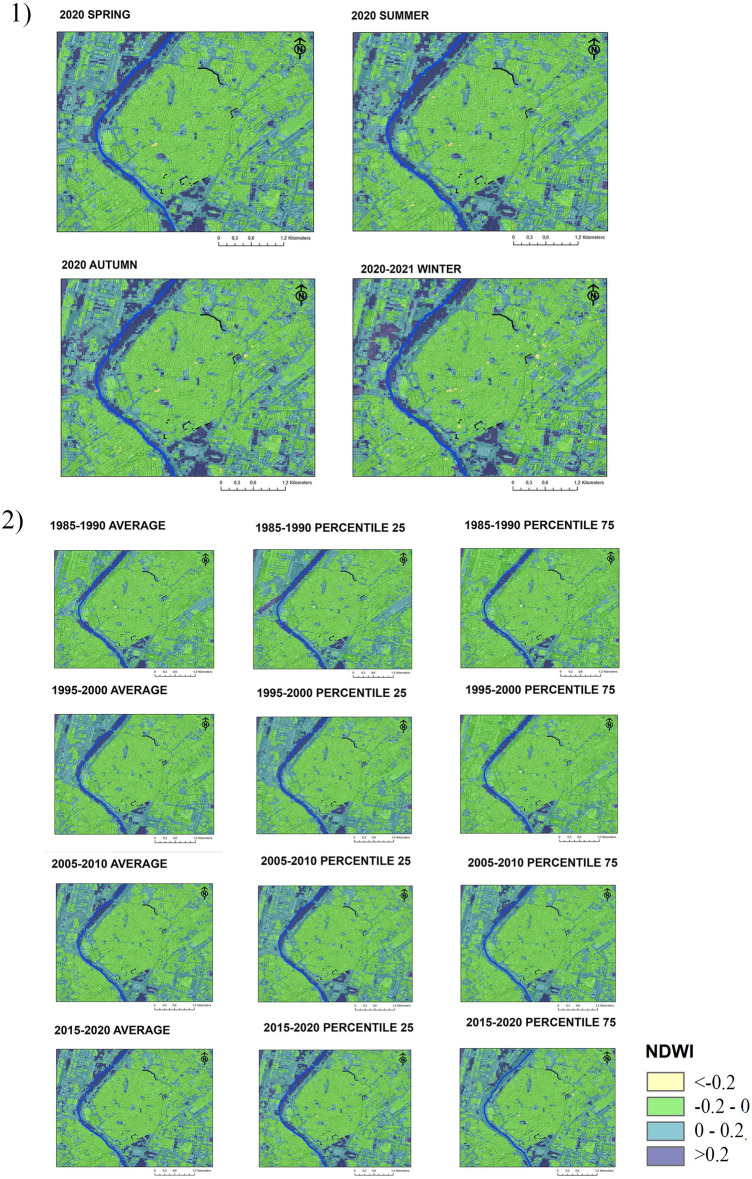
NDWI maps of Seville. (1) Seasonal averages in 2020 and 2021; (2) Five-year averages and percentiles in 1985–1990, 1995–2000, 2005–2010, 2015–2020. The fortifications are represented in black lines and the river channel in blue.

In the case of Seville, Fig. 6 shows the non-existence of seasonal fluctuations in humidity throughout 2020–2021. The points of highest humidity remain constant with hardly any changes between winter and summer. Unlike Niebla, the city of Seville does record areas with NDWI levels above 0.2. Associated with urban gardens, some structures of the fortifications analysed are located in many of these points of higher. The most significant example is the Reales Alcázares gardens (urban sections 3 and 4, according to figure 2), located on the south side of the historic centre and identified on the maps as one of the greatest vegetation and moisture points in the city.

Figure 6 shows a gradual increase in NDWI levels in the urban areas surrounding the historic centre of Seville since 1995. The wettest points are those near the banks of the Guadalquivir river and those in which there has been a development of landscaped areas. The most significant points are the expansions to the south eastern side and the north western side of the city.

## Vegetation monitoring at specific points using functions

**Figure 7:**
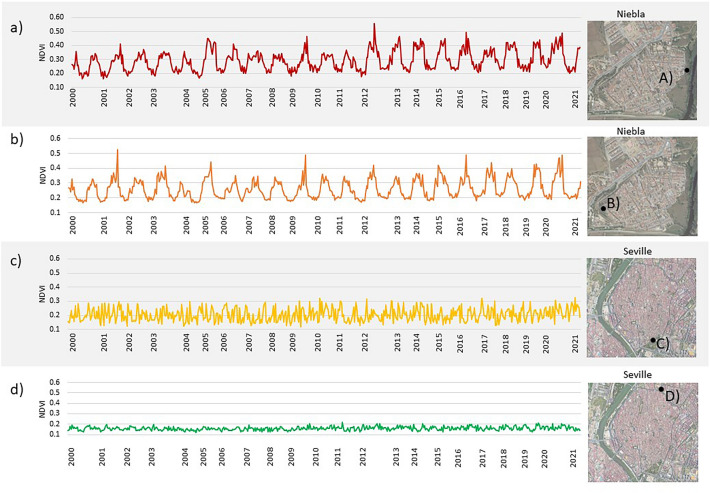
NDVI values since 2000 according to different vegetative coverage: (**A**) Natural River Forest in Niebla; (**B**) Semi-natural forest in Niebla; Historic Garden in Seville; (**D**) Irrigated grassland in Seville. Color indicates level of risk according to dense vegetation, fluctuation and humidity levels (red: high hazard, green: low hazard).

Figure 7 explains the results of monitoring the changes identified in the NDVI of different urban vegetation covers since 2000 using functions and satellite images. Figures 7.A and 7.B. show peri-urban forest in Niebla, where the areas with the highest values of both NDVI and NDWI are located. Besides, these are the points most affected by seasonal fluctuations marked by the phenological cycles of Mediterranean vegetation. While these cycles can differ depending on the species and location, they generally involve active growth in the spring and a halt in growth during the summer and winter. Despite some areas having access to subsurface water during the summer months (Figs. 5 and 6), the harsh Mediterranean summer climate significantly restricts vegetation growth in these regions. For these reasons, these points are clearly discernible as the points of greatest hazard for historic rammed earth fortifications.

In areas in which irrigation is controlled such as historic gardens (Fig. 7C) and parks (Fig. 7D) the NDVI levels recorded are lower and stable annually. Dense historic gardens as Reales Alcazares garden in Seville, show levels of NDVI near to 0.3. The NDWI levels are also slightly higher than those of bare soil and range between 0.06 and 0.13, yielding values of up to 0.22. Since 2018 there has been a slight increase in the minimum annual values, which indicates despite the characteristics of the Mediterranean climate and the droughts, this historic garden is increasing vegetative density also during the summer. In parks and grassland NDVI values are between 0.15 and 0.20 with stable annually levels, which do not pose a risk to the conservation of earthen historic walls.

On the other hand, the paved areas in the cities analysed have NDVI levels below 0.2 This coverage is only observed in the surrounding of a few of the Seville fortifications as the walls in Callejon del Agua (urban section 3 according to figure 2) and Cabildo square (urban section 5 acoording to figure 2). These levels of NDVI correspond to soils that do not present a threat due to vegetative development but in case of intense storms, they can pose a risk due to the difficulties of acting as buffer zones and drainage of the fortifications^62^.

## Discussion: green areas in sustainable historic fortified cities

Promoting sustainable development in historic cities requires to implement measurement and protocols that foster a balance between environment, society, and economy. In this regard, preserving cultural heritage and creating green spaces are key aspects for the sustainable management of historic cities. Encouraging their maintenance and utilization as urban resources not only improves the quality of life for residents but also promotes tourism and local economy, while reducing negative environmental impact in the city^73–75^.

The study shows the urban planning policies developed in Niebla and Seville have favored the implementation of the precepts of cultural sustainability promoted by UNESCO and ICOMOS. Both cities show an increase in the surfaces occupied by green areas since 1985. These data are associated with decrease in the population of Seville (701,927 inhabitants in 1995 and 684,234 inhabitants in 2020) and an increase in the population of Niebla (3,846 inhabitants in 1995 and 4,158 inhabitants in 2020). Despite the urbanization processes in Niebla, the existing pressure on green areas has not increased.

The observed trend coincide with the studies carried out by Kabisch and Haase that indicate a general increase in green areas in South and West European Cities^76^; and Fuller and Gastón^77^ that associate the increase in green areas with the availability of urban spaces, not with the population changes. From this perspective, the presence of historic fortifications and buffer zones without buildings minimizes the compactness of the city and works as a driver to promote the development of green areas, and more sustainable and resilient cities. This trend towards the development of more sustainable cities presents large gaps between the cities of Eastern and Western Europe. Easter European cities show immersed processes with urban densification processes^78^ and a decrease in green areas since 2005^76^.

Furthermore, the coexistence of natural resources and cultural heritage in historic cities makes them unique spaces from which to develop urban planning policies that support the achievement of Sustainable Development Goals (SDGs) related to SDG 11 (Sustainable Cities and Communities), SDG 13 (Climate Action), and SDG 3 (Good Health and Well-being). In historic cities, cultural heritage is a source of inspiration and connection with the history and identity of the community. The combination of green areas and heritage spaces promotes outdoor recreation, improves physical health, enhances citizen well-being, and fosters social cohesion through the communal use and learning of heritage resources^74,75,79^.

The urban interventions carried out during the last 30 years in Seville and Niebla use historic fortification buffer zones to enable green areas that contribute to the sustainability and habitability of historic cities. In fact, green areas are the most frequent land cover in the immediate surroundings of the fortifications (NDVI 0.2–0.5). Most of them are public gardens and parks, elongated and located next to paths and sidewalks. The increase in green spaces reflects an interest in generating mixed areas, and comprising green and heritage landscapes that promote walkability next to the fortifications^80,81^. In practice, the increase of urban green areas is beneficial to minimize urban noise, pollution, effects of climate change, and urban heat islands^82,83^. Besides, green areas have a direct protective function of the fortifications because vegetative cover minimizes the damage caused by impermeable ground such as asphaltic in which earthen fortifications act as an outlet for the waters contained in the subsoil.

In turn, no direct relationship has been observed between the presence of green urban areas and the increase in humidity above risk levels (NDWI > 0.2). In some specific point of Niebla fortifications (urban Sect. 9) the increase of humidity, and fluctuations in the density of vegetation could favor the development of pathologies such as moist areas, efflorescence, iron rich patinas, detachments and erosion in earth rampart 91], [92]^65,85^. In this situation, monitor the NDVI and NDWI levels identify the growth and density of vegetation, and the humidity level, making possible to formulate hypotheses regarding the exposure to danger of the different walls and how this can affect their durability. In hight humidity areas, it is essential to minimize the vulnerability of the fortifications, monitor the state of conservation, and restore the most deteriorated walls that can favor the access of humidity to the interior of the wall, erosion processes and collapses. The characterization of construction materials^86–88^ joined to the use of vulnerability indices and GIS^28,65,84^ are useful instrumentsto evaluate the risk exposure of the fortifications in these contexts. The diagnosis model developed by Moreno et al.^65^ for earth fortifications offers tools for on-site diagnosis and vulnerability index that allow gathering information about the presence of pathologies caused by the direct impact of vegetation on walls or roots in wall foundations. These resources permit to identify affected areas, the frequency of occurrence, and the intensity of damage. Furthermore, they help to establish the correlation between this information, moisture levels and vegetation health data obtained through satellite images.In the studied cities all the rammed earth walls located in paved areas have a perimeter of bare ground, which minimizes the effects of the asphalt surrounding it and permits the evapotranspiration from the subsoil. This shows an intentionality of the urban interventions to preserve the fortifications generating unpaved spaces that favors permeability and minimize damage due to water access from the subsoil.

To sum up, the present study puts forth a tool that enables monitoring the impact of green areas in the vicinity of earthen fortifications. The inclusion of the aforementioned method in the preventive conservation plans of fortifications is of paramount importance in order to assist historic cities to implement sustainable urban policies that foster the development of green spaces and the conservation of open-air heritage resources. Currently, one of the primary challenges for historical cities is evaluating whether the measures implemented in heritage environments effectively contribute to attaining the SDGs and promoting sustainable education^89–91^. The lack of comprehensive methods for data analysis, the scarcity of tools that promote digital transition, and the lack of social awareness are the main problems highlighted in the literature^92,93^. In response to this need, the proposed method demonstrates significant potential to analyse the synergies between green areas and architectural heritage, while also promoting the implementation of effective and sustainable measures that yield greater well-being for the population and environmental improvements.

However, it is worth noting that the analysis only covered historic cities located in Mediterranean climates. To validate the representativeness of the findings, future research would require to replicate the proposed method in different climatic zones and green spaces.

## Conclusions

In conclusion, this study has demonstrated a growing trend in the inclusion of green areas in historic cities such as Seville and Niebla, and how these areas are influencing the conservation of their earthen fortifications. Regarding to the conservation of architectural heritage, the urban parks and historic gardens analysed present health vegetation (NDVI 0.2–1) and low levels of humidity (NDWI < 0.2) that do not pose a risk for the conservation of the historic earthen fortifications.

The tested method to analyse series of images improves the reliability data and favors the identification of normal values and periods with higher and lower humidity-vegetation. Besides the use of reducers and functions allows to monitor the changes that have occurred in the last 30 years and the monthly, annual and interannual variability.

In short term, this methodology facilitates access to useful satellite resources and favour a better understanding of the effect of green areas on the durability of earthen fortifications. In the long term, these type of studies favour the comprehensive monitoring of green areas and humidity near to heritage building facing situations of anthropic-climate change and/or emergency.

Overall, this study highlights the importance of monitoring and conserving the earthen heritage located in green areas in historic cities. The application of this methodology can help to develop more respectful and sustainable heritage management policies within the urban environment.

## Supplementary Information

Supplementary Information.

Supplementary Information 1.

## Data Availability

The datasets generated and/or analysed during the current study are available in the Google Earth Engine repository, https://developers.google.com/earth-engine/datasets/. The images obtained with the means and percentiles of the series of satellite images can be elaborated from the scripts included as supplementary material to the article.
