# Supplementary material for: Analysis of the impact of green urban areas in historic fortified cities using Landsat historical series and Normalized Difference Indices
Source: Sci Rep. 2023 Jun 2;13:8982. doi: 10.1038/s41598-023-35844-8 (PMC10238395; doi:10.1038/s41598-023-35844-8)
Supplement: Supplementary file 1 — Supplementary Information. [file 41598_2023_35844_MOESM1_ESM.pdf]

This link provides access to the Landsat satellite image collection and allows you to replicate the proposed methodology for calculating NDVI in the article titled "The effects of green urban areas in historic fortified cities: an analysis by Landsat historical series and Normalized Difference Indices". The scripts that you will find in this link have been developed as part of this article. Please remember to cite the article if you use them in other analyses. Besides, to use it, you must have a Google Earth Engine account.

https://code.earthengine.google.com/?accept_repo=users/monicamorenofalcon1983/NDVI
